# Oral propranolol for the treatment of hemangiomas in high-risk infants: safety and cost analysis of outpatient-initiated therapy

**DOI:** 10.3389/fmed.2024.1439449

**Published:** 2024-09-13

**Authors:** Rina Su, Hua Qian, Cui Hu, Wei Li, JiBin Li, Bo Wu, Yang Gu, Ting Zhang, YaFen Wu, YingYing Qian, Hui Lu

**Affiliations:** ^1^Department of Dermatology, Beijing Chao-Yang Hospital, Capital Medical University, Beijing, China; ^2^Department of Dermatology, Children’s Hospital of Soochow University, Suzhou, Jiangsu, China

**Keywords:** infantile hemangioma, outpatient treatment, safety analysis, cost analysis, oral propranolol

## Abstract

**Objectives:**

To investigate the safety and cost analysis of oral propranolol treatment for high-risk infantile hemangiomas starting from the outpatient setting.

**Methods:**

A total of 41 high-risk infantile hemangioma patients from outpatient settings and 43 from inpatient settings were selected for the study. After routine pre-treatment examinations, patients were administered propranolol in a stepwise incremental dosing regimen over three consecutive days in the outpatient clinic. Changes in heart rate, blood pressure and PR interval before and after medication were compared. On the 10th day post-medication, liver and kidney functions, fasting blood glucose, tumor ultrasonography, and electrocardiogram were re-evaluated. The costs of treatment starting from the outpatient clinic (including pre-treatment examinations and the first three days of treatment) were calculated and compared with those of similarly managed inpatient cases.

**Results:**

The majority of patients exhibited a reduction in heart rate and blood pressure, as well as an extended PR interval after treatment of medication (*P* < 0.05), which remained within normal limits without clinical symptoms. On the 10th day post-medication, statistical differences in blood biochemistry and electrocardiograms were observed when compared to pre-treatment values (*P* < 0.05), but all values remained within normal ranges. No severe adverse reactions such as hypoglycemia occurred. Additionally, the cost of treatment from the outpatient clinic was significantly lower than that of inpatient care.

**Conclusion:**

Oral propranolol treatment for high-risk infantile hemangiomas starting from the outpatient setting is associated with few adverse reactions and significantly reduced treatment costs. It is worthy of broader application in hospitals without dermatology wards.

## 1 Introduction

Infantile hemangiomas (IH) are the most common benign skin tumors in infancy, with an incidence rate of approximately 1.5–12%, and there appears to be an increasing trend over the years (1, 2). In recent times, propranolol has emerged as the first-line treatment option for high-risk IH, with its safety and efficacy widely recognized (3, 4). Traditionally, hospitals administer oral propranolol as an inpatient treatment, which is more costly and burdensome for parents, and carries a risk of nosocomial infections. Research has shown that propranolol treatment for infantile hemangiomas has both advantages and disadvantages in outpatient and inpatient settings (5–7). Although propranolol is effective, experts have not reached a consensus on the optimal treatment setting.

To investigate the safety and cost of initiating oral propranolol treatment for IH in an outpatient setting, a study was conducted with 41 pediatric patients from our dermatology outpatient clinic. The study analyzed the safety of a three-day step-up dosing regimen of oral propranolol and calculated the costs associated with outpatient treatment initiation (including pre-treatment examinations and the first three days of treatment). These costs were then compared with those incurred under inpatient treatment conditions for similar cases.

Our analysis seeks to provide evidence-based insights into the efficacy, safety, and overall outcomes of propranolol treatment in different clinical environments. This research could potentially guide healthcare providers in making informed decisions regarding the optimal treatment setting for infantile hemangiomas, ultimately improving patient care and resource allocation.

## 2 Materials and methods

### 2.1 Clinical data

A selection of 41 and 43 pediatric patients treated at the Dermatology Outpatient and inpatient of the Children’s Hospital affiliated with Soochow University from June 2016 to December 2017 was studied. Inclusion criteria for the study are as follows: (1) Rapidly growing large-area hemangiomas with typical clinical manifestations, supported by relevant imaging results such as ultrasound, and in some cases, CT and MRI; (2) Hemangiomas located in special areas such as around the eyes, mouth, or nose; (3) Hemangiomas that have developed ulcers; (4) Hemangiomas classified as high-risk IH according to the consensus on the treatment of IH with β-Blockers by Chinese Experts (8); (5) Presence of six or more multiple hemangiomas; (6) No prior interventions before medication administration. Exclusion criteria include: (1) Allergy to propranolol; (2) Children with pneumonia, bronchitis, bronchial asthma, liver dysfunction, renal impairment, thyroid disorders, hypotension, atrioventricular block, or sinus bradycardia, which could impede treatment (9).

### 2.2 Treatment protocol

#### 2.2.1 Ethics approval and consent to participate

The study has received the approval of the Ethics Committee at Children’s Hospital of Soochow University. Prior to administering medication to all children, guardians are thoroughly informed about the potential risks associated with the medication and the contingency plans for risk management. Informed consent is obtained by signing a consent form.

#### 2.2.2 Medication administration

The oral dose of propranolol is gradually increased over the first three days according to the following dosages: 0.5, 1, 1.5–2 mg/kg/day, divided into three doses. According to the Chinese Expert Consensus and related guidelines, we conducted comprehensive monitoring throughout the entire course of propranolol treatment for infantile hemangioma (8, 10–14). This included thorough assessments for all selected children, including blood and urine tests, liver and kidney function tests, myocardial enzyme spectrum analysis, a complete thyroid function panel examination, fasting blood glucose measurement, echocardiography with Doppler imaging, electrocardiogram (ECG) recording, and an upright chest X-ray. The evaluation time points were established at pre-treatment, followed by assessments at 1, 2, and 3 h post-treatment. Additionally, follow-up evaluations were conducted at 10 days and 1 month after the treatment.

#### 2.2.3 Educating parents on oral propranolol

Parents should ensure their child’s heart rate stays above 90 beats/min during calm sleep and 100 beats/min during regular activity. Administer medication after meals, especially for demand-fed infants, to avoid hypoglycemia. Monitoring the child’s mental state and sleep is vital. If an infection or inflammation occurs, halt medication temporarily and resume after recovery. Gradually taper off medication rather than stopping suddenly. Continual follow-ups are necessary post-treatment. Parents should regularly photograph the tumor and show these images to the doctor during visits. Seek immediate medical help for severe complications.

## 2.3 Statistical analysis

Statistical analysis is performed using SPSS18.0 software. Count data are represented as (mean ± SD). The *p*-value is calculated using the *t*-test method, with *P* < 0.05 indicating statistically significant differences.

## 3 Results

### 3.1 Demographic data of inpatient and outpatient patient

The outpatient IH cohort consisted of 27 female and 14 male infants, with ages ranging from 2 to 12 months, and an average age of 5.3 ± 1.4 months. The locations of the hemangiomas were as follows: 21 cases on the head and face, 9 on the trunk, and 11 on the limbs. 43 infants receiving inpatient treatment met the inclusion criteria, including 33 female and 10 male infants, with ages ranging from 2 to 15 months and an average age of approximately 4.8 ± 1.5 months. The locations of the hemangiomas were as follows: 19 cases on the head and face, 15 on the trunk, and 9 on the limbs (Table 1).

**TABLE 1:** Demographic data and medical cost of inpatient and outpatient patients.

		Inpatient (*n* = 43)	Outpatient (*n* = 41)
Age (month)		4.8 ± 1.5	5.3 ± 1.4
No. of females		33	27
Location	Head and face	19	21
	Trunk	15	9
	Limbs	9	11
Hospital stay (days)		4.8	0
Medical cost (RMB)		3,383.2	795.7*

*p < 0.05.

### 3.2 Safety analysis

**FIGURE 1:**
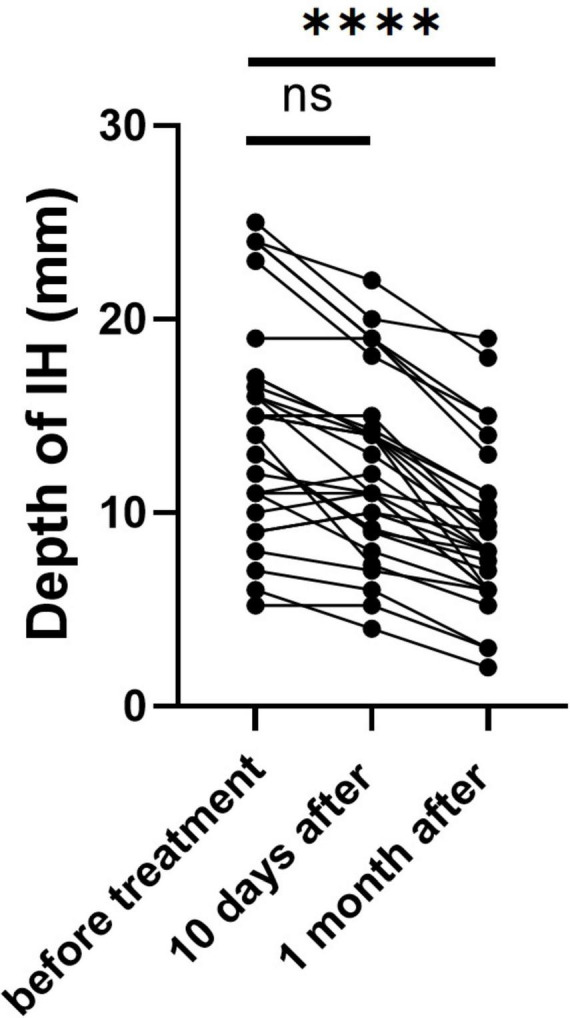
Depth of IH before and after medication. ****p < 0.0001.

We utilized ultrasound to measure the depth of hemangiomas. The treatment led to a significant reduction in hemangioma depth compared to baseline measurements. Initially, the average depth of the hemangiomas was 14.49 ± 5.43 mm. After ten days of treatment, this depth decreased to 12.34 ± 4.57 mm. Further reduction was observed one month post-treatment, with the average depth decreasing to 9.01 ± 4.07 mm (*p* < 0.0001) (Figure 1). During the initial three days of medication administration, pediatric patients’ heart rates, blood pressure, and ECGs were meticulously monitored both pre- and post-administration. The results indicated a statistically significant reduction in heart rate and blood pressure, as well as a prolonged PR interval (*P* < 0.05) (Table 2). These changes, however, remained within the normal range and did not manifest any clinical symptoms. The study monitored PR interval prolongation over the initial three days of treatment, recording measurements of 17.58 ± 19.88 ms on the first day, 11.39 ± 22.35 ms on the second day, and 4.92 ± 19.52 ms on the third day. Importantly, the data revealed no significant correlation between the dosage administered and the degree of PR interval prolongation during this early treatment phase. Two children exhibited asymptomatic bradycardia one hour after the first dose. An urgent ECG showed sinus bradycardia with prolonged PR interval, but no special treatment was given. Two hours later, the heart rates of these children returned to normal, and a follow-up ECG showed no significant abnormalities. On day 10 post-medication, blood tests showed statistically significant differences in fasting blood glucose and liver and kidney function compared to pre-treatment (*P* < 0.05), yet they were still within normal limits, with no incidents of severe adverse reactions such as hypoglycemia (Table 3). Out of the 41 selected pediatric patients, one discontinued treatment on day 5 due to intolerance of gastrointestinal reactions, which improved without special treatment; one was lost to follow-up; three ceased treatments due to strong opposition from elders worried about side effects. No other notable adverse reactions were observed. Therefore, these five cases were excluded from subsequent analysis.

**TABLE 2:** Heart rate (beats/min), blood pressure (mmHg) and PR interval (ms) changes before and after medication.

		Before	After
Heart rate	1st day	135.92 ± 6.98	121.81 ± 8.86*
	2nd day	130.53 ± 8.88	115.44 ± 8.50*
	3rd day	127.39 ± 9.88	118.78 ± 7.22*
Systolic pressure	1st day	87.94 ± 7.77	80.42 ± 6.41*
	2nd day	84.92 ± 7.56	79.97 ± 5.40*
	3rd day	82.31 ± 6.15	79.78 ± 5.61
Diastolic pressure	1st day	40.28 ± 3.00	36.58 ± 2.45*
	2nd day	38.08 ± 2.33	36.47 ± 1.80*
	3rd day	37.00 ± 2.04	35.92 ± 1.91*
PR interval	1st day	104.61 ± 15.93	122.19 ± 12.20*
	2nd day	106.89 ± 18.86	118.28 ± 7.56*
	3rd day	110.61 ± 17.87	115.53 ± 5.74

*p < 0.05.

**TABLE 3:** Fasting blood glucose and liver and kidney function before and after 10 days of medication treatment.

	Before	After
Fasting blood glucose (mmol/L)	4.92 ± 0.32	4.75 ± 0.27*
BUN (mmol/L)	3.53 ± 0.93	3.79 ± 0.75*
Cr (μmol/L)	23.32 ± 4.76	22.31 ± 3.84*
AST (U/L)	36.93 ± 7.15	39.14 ± 7.03*
ALT (U/L)	25.25 ± 5.79	26.16 ± 5.92*

*p < 0.05.

### 3.3 Cost analysis

We selected 41 and 43 pediatric patients treated at the Dermatology Outpatient and Inpatient departments for the cost analysis according to our inclusion and exclusion criteria. In this study, we focused solely on direct medical costs, which include physician fees, hospital charges, medication costs, and diagnostic tests. This focus is primarily because we aim to assess the direct economic impact of treatment to inform medical decision-making. Additionally, direct medical costs have higher data availability and lower measurement complexity, ensuring data accuracy and reliability.

Outpatient treatment for infantile hemangiomas is significantly more cost-effective when compared to the expenses incurred for inpatient care under similar conditions. The average length of hospital stay for these selected patients was 4.8 days, with an average cost of RMB 3,383.2. In contrast, our analysis of outpatient treatment costs showed that including pre-treatment examinations, the infants visited the hospital for four mornings (totaling about 20 h), with an average cost of RMB 794.8 (Table 1).

## 4 Discussion

In 2008, French physician Léauté-Labrèze and colleagues accidentally discovered that oral administration of propranolol tablets could effectively treat infantile hemangiomas (IH) (4). Subsequent research from around the world has delved into the efficacy, safety, and mechanisms of action of oral propranolol for treating IH. IH in high-risk groups tend to grow rapidly and are prone to serious complications such as necrosis, ulceration, and even blindness or disfigurement, necessitating aggressive treatment. Propranolol offers significant advantages in treating high-risk IH. It effectively controls the rapid growth of these vascular tumors, thereby reducing the risk of associated complications. By initiating timely treatment, propranolol helps minimize the potential for long-term skin changes and functional impairments that IH can cause. Additionally, for cases where IH may result in permanent skin alterations or impact the function of critical organs, propranolol therapy can significantly improve the patient’s long-term prognosis (4, 11, 15).

The administration of propranolol for infantile hemangiomas has been demonstrated to possess both advantages and disadvantages in outpatient and inpatient settings (5–7). Traditionally, outpatient treatment has been considered more cost-effective and convenient, allowing patients to remain in a familiar environment without significant adverse events reported. This approach is typically used for patients with milder symptoms. However, inpatient treatment is recommended for high-risk groups, such as very young infants or those with preexisting conditions, due to the need for close monitoring of blood pressure and heart rate. Inpatient settings also allow for controlled dosing and observation. In this study, we utilized data from the dermatology department of a tertiary children’s hospital in China to address this issue. We compared the effectiveness, safety, and cost-effectiveness of outpatient versus inpatient treatment for high-risk infantile hemangiomas. Our findings suggest that with adequate management and monitoring, outpatient treatment can be both safe and effective while also being economically advantageous for high-risk patients.

In countries outside of China, for children older than eight weeks, treatment typically begins on an outpatient basis, with an initial dose of 1–2 mg/(kg⋅d), administered in three divided doses (15, 16). This dose is maintained for 3–7 days to ensure tolerance before increasing the dosage. The target dose of 2–3 mg/(kg⋅d) is usually reached within 1–2 weeks, with an efficacy rate of over 90% (17, 18). The approach to medication in China is similar to that of other countries. However, due to the lower plasma binding rate and clearance rate of propranolol in Asians compared to Caucasians (19, 20), the starting dose is typically reduced to 0.5 mg/(kg⋅d), with a target dose ranging from 1.5 to 2 mg/(kg⋅d). However, most hospitals in China require patients to be hospitalized and monitored by electrocardiogram (ECG) when starting the medication. Given the current limitations of oral propranolol treatment for Infantile Hemangiomas (IH), such as the extended time required to increase the dose and the need for hospitalization and monitoring which can be complicated, a new outpatient-based method of administering medication over three days in a stepwise manner has been adopted. This method aims to explore the feasibility of initiating treatment from an outpatient setting in actual clinical practice.

Approximately 75% of adverse reactions associated with oral propranolol treatment for IH manifest within the initial month of initiating therapy. In a study involving 1,260 pediatric patients, significant side effects were observed in 26 cases (2.1%). Among these cases, severe sleep disturbances were reported in 17 cases (65.3%), while respiratory illnesses occurred in 4 cases (15.3%) (21). Another study included a cohort of 220 children indicating no severe treatment-related adverse events, although 27 patients experienced minor side effects. There was a significant decrease in heart rate following each of the first two doses (*p* < 0.001) and a notable reduction in systolic blood pressure on day 2 at a dosage of 1 mg/kg/day after the first dose (*p* = 0.01). Blood glucose levels remained stable throughout the treatment period (13). Other common adverse reactions included gastrointestinal discomfort, cold extremities, as well as asymptomatic bradycardia, hypotension, and hypoglycemia (21, 22). In our own study, we observed transient bradycardia in 2 children, which normalized after 2 h upon re-evaluation; one patient experienced reduced appetite and diarrhea as gastrointestinal reactions, which resolved after cessation of treatment and showed no abnormalities upon follow-up. The majority of the children had heart rates reduced to 95–125 beats per minute, and monitoring of biochemical markers and mental status revealed no significant abnormalities. Our findings suggest that oral propranolol treatment for high-risk group IH does not necessarily require hospitalization and can be considered for outpatient initiation. Most adverse reactions can return to normal without special intervention, and starting or increasing doses in an outpatient setting is also safe.

A cost analysis study from the Cleveland Clinic Lerner College of Medicine revealed that the average cost of inpatient treatment for infantile hemangiomas using oral propranolol was $2,603 per day, which increased to $2,843 per day when an echocardiogram was included. The anticipated cost for outpatient treatment was significantly lower at $138 per day, rising to $828 per day with the addition of an echocardiogram (6). Our findings are consistent with these results, suggesting that initiating treatment on an outpatient basis, taking into account pre-medication evaluation and the initial three days of medication, entails significantly lower costs compared to inpatient care under similar circumstances. By implementing an incremental approach spanning three consecutive days, we successfully minimized the time intervals, thereby significantly reducing both the financial expenses associated with treatment and the temporal burden on patients’ families.

The findings from the study indicate that administering oral propranolol for the treatment of infantile hemangioma in an outpatient setting, while monitoring pertinent health indicators, is a viable approach. The adoption of an expedited dosage escalation protocol, achieving the target dose within a three-day period, has been demonstrated to be both safe and efficacious. Moreover, this method offers the added benefit of reducing the financial burden of treatment. As such, it presents a considerable opportunity for healthcare facilities, particularly those lacking specialized dermatology departments, to enhance patient care. Nonetheless, it is important to acknowledge that the study’s conclusions are drawn from a relatively small cohort of cases. Consequently, to substantiate these findings, further investigations involving a more substantial number of participants are warranted.

## Data Availability

The raw data supporting the conclusions of this article will be made available by the authors, without undue reservation.
